# A Century of Bacteriophages: Insights, Applications, and Current Utilization

**DOI:** 10.3390/antibiotics14111080

**Published:** 2025-10-27

**Authors:** Sadika Dkhili, Miguel Ribeiro, Karim Ben Slama

**Affiliations:** 1 Laboratoire de Recherche “Bioressources, Environnement et Biotechnologie (BeB)” LR22ES04, Institut Supérieur des Sciences Biologiques Appliquées de Tunis, Université de Tunis El Manar, Avenue Docteur Zouheir Essefi, Tunis 1006, Tunisia; sadikadkhili@yahoo.com; 2 Department of Genetics and Biotechnology (DGB), University of Trás-os-Montes and Alto Douro (UTAD), 5000-801 Vila Real, Portugal; 3 Chemistry Research Centre (CQ-VR), Food and Wine Chemistry Lab., University of Trás-os-Montes and Alto Douro (UTAD), 5000-801 Vila Real, Portugal

**Keywords:** bacteriophages, health therapy, antimicrobial resistance, water treatment, environmental remediation, food safety

## Abstract

Bacteriophages or phages are viruses that exclusively target and replicate within bacteria, acting as natural predators in the biosphere. Since their discovery over a century ago, host-specific bacteriophages have been widely advocated as a cost-effective and adaptable approach to controlling and combating bacterial infections. Antibiotic resistance, a growing concern and a significant global public health problem, has further underscored the importance of bacteriophages. Nevertheless, their potential applications span diverse fields, including molecular biology, phage therapy, bacterial detection, food safety, and wastewater decontamination. Furthermore, bacteriophages represent a diverse group of viruses that are relatively easy to handle, making them suitable for use in both treatments and biotechnology research. In this review, we aim to provide a comprehensive overview of bacteriophage history, characteristics, and applications that have been employed to address human challenges, ranging from healthcare to environmental remediation. We will highlight key findings and outcomes, shedding light on ongoing research that will shape the future of bacteriophage applications.

## 1. Introduction

**Figure 1:**
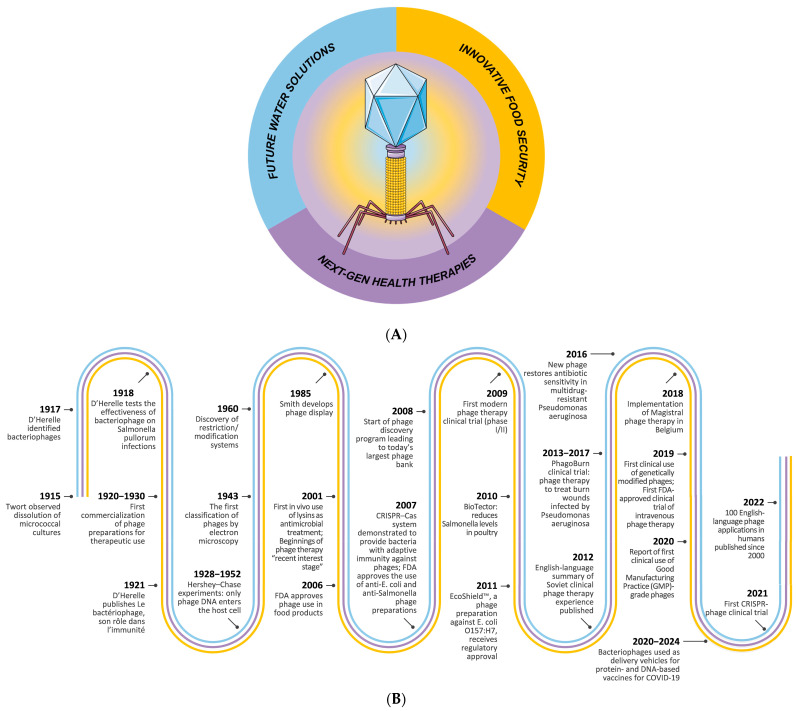
Graphical overview of promising applications of phages (**A**) and timeline of major phage events (**B**).

Bacteriophages or phages are viruses that infect only bacteria. They are obligate intracellular parasites [1]. They are the most abundant biological entities on the planet, with an estimated total population of over 10^31^ phages [2], being present in oceans, lakes, sewage, drinking water, soil and food [3]. They were co-discovered independently by Frederick William Twort and Félix Hubert d’Hérelle [4] (Figure 1B). In 1915, Twort noticed that there was a filterable, transparent, and infectious agent that induces the dissolution of cultures of micrococci, without defining its nature.

Subsequently, in 1917, d’Hérelle became interested in an epidemic of dysentery. By testing the stool filtrates of patients, he found that the turbidity of the bacterial culture had disappeared giving way to a clear solution after an overnight incubation. He deduced that it was probably an invisible microbe, a parasite of bacteria. He gave it the name of bacteriophages [4]. The first commercialization of phage preparations was started in 1928 in France by the Robert and Carrière laboratories. During the 1930s, the ten best-selling drugs were reserved for the five phage-based specialties (bacté-intesti-phage; bacté-coli-phage; bacté-rhino-phage; bacté-pyo-phage; bacté-staphy-phage) [5]. The appearance of the electron microscope supported the establishment of the first classification of phages in 1943 by Ernst Ruska. So far, more than 5500 phages have been examined by electron microscope [6,7].

**Figure 2:**
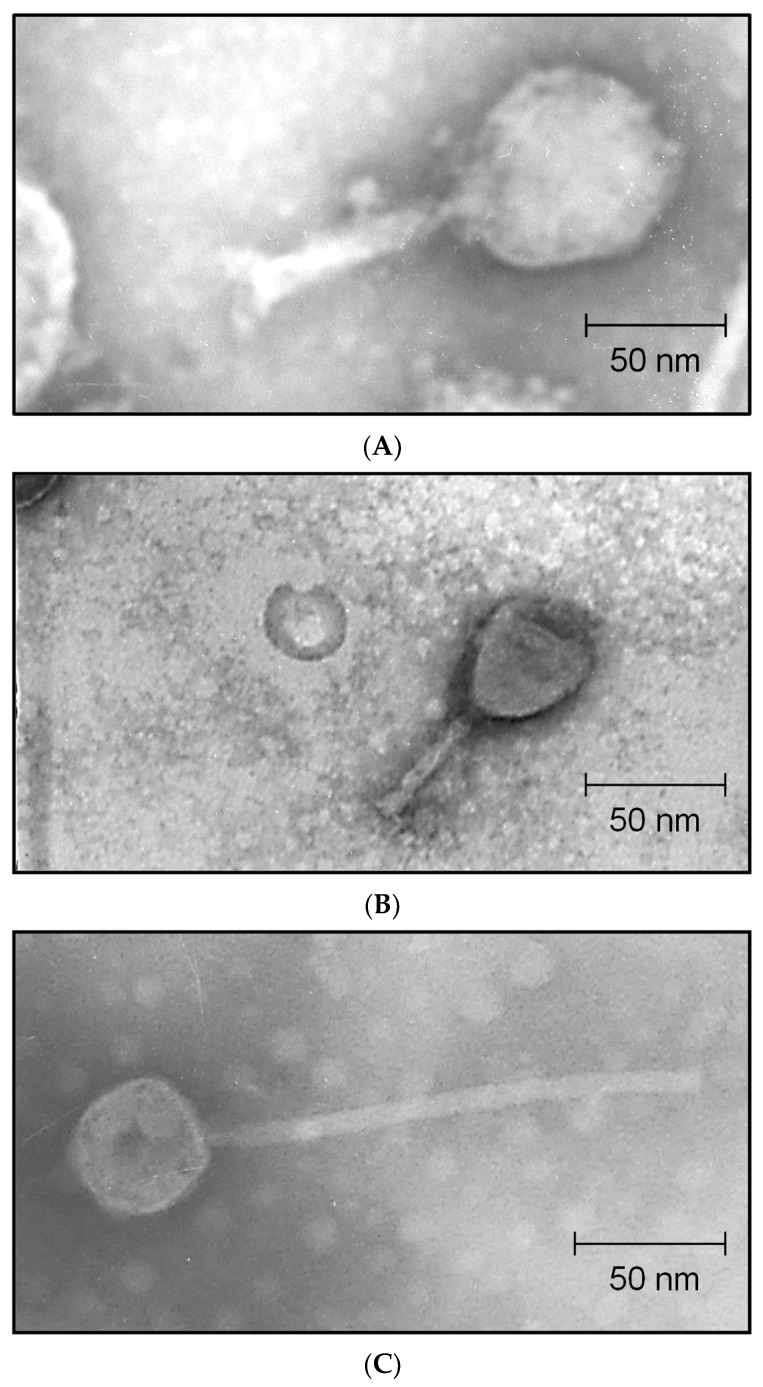
Some transmission electron microscopy images of *Escherichia coli* (**A**,**C**) and *Enterobacter* (**B**) phages. The phage in (**A**,**B**) exhibited a contractile tail (42 ± 3 nm, 50 ± 2 nm) and an icosahedral head (82 ± 2 nm, 65 ± 2 nm), respectively, a morphology characteristic of the former *Myoviridae* family. The phage in (**C**) is characterized by a circular head (69 ± 6 nm) and a long, non-contractile tail (217 ± 8 nm), a morphology characteristic of the former *Siphoviridae* family. All images presented in this figure were acquired by the authors.

The phage taxonomy is regulated by the International Committee on Taxonomy of Viruses (ICTV), which classifies viruses across several hierarchical levels, including class, order, family, subfamily, genus, and species [8]. As new bacteriophages continue to be identified, the ICTV regularly updates its taxonomic framework. In the most recent revision (August 2022), the ICTV restructured the phage classification system, discontinuing several long-established families such as *Siphoviridae*, *Podoviridae*, and *Myoviridae*. These revisions, which reorganized many tailed phages under the class *Caudoviricetes*, have notable implications for phage family-level classification and comparative analyses. They have very diverse morphologies, including long non-contractile tails, short non-contractile tails, contractile tails, and even atypical or tail-less forms. Figure 2 shows some transmission electron microscopy images of *Caudoviricetes* phages infecting *Escherichia coli* and *Enterobacter* as examples of this morphological diversity.

Phages display extensive variation in structural dimensions, particle morphology, and genomic organization [6,9,10]. They have a variety of structural morphologies, among which tailed double-stranded deoxyribonucleic acid DNA (dsDNA) phages are the most abundant [8]. Besides (dsDNA) phages, there are also phages with single-stranded DNA (ssDNA) [11], single-stranded ribonucleic acid RNA (ssRNA) [12] or double-stranded RNA (dsRNA) [13,14]. *Caudoviricetes* phages, which are tailed double-stranded DNA viruses, have evolved over extensive periods, allowing them ample time to diversify. This diversification has led to the accumulation of point mutations, intragenomic rearrangements—including deletions, duplications, inversions, and transpositions—and frequent recombination events [15,16]. In their experiments, Hershey and Chase analyzed the phenomenon between phages and bacteria during viral infection [17].

As early as the 1950s, scientists discovered that when bacteriophages infected a host bacterium, they first attached themselves to the outside of the bacterium. Then, a fragment of the phage entered the bacterium and replicated inside the cell. Lysis, or bursting, of the host bacterium is caused after numerous replications, thus killing the host bacterium. Scientists classified this replicated fragment as genetic material [17]. The restriction–modification (R-M) system was discovered through experiments in the 1950s involving bacteriophages and bacteria. Researchers observed that some bacterial strains were resistant to phage infection, and this resistance was linked to a bacterial defense mechanism that involved modifying and restricting phage DNA. This led to the discovery of restriction enzymes, which cut DNA at specific sequences, and modification enzymes (methyltransferases), which protect bacterial DNA by adding methyl groups to these sequences [18]. In Belgium, phage therapy is implemented through magistral preparations, which allows pharmacists to process natural phages as active ingredients in customized medications.

This approach, facilitated by a specific legal framework, enables the use of phages to treat bacterial infections, particularly in cases where conventional antibiotics are ineffective. Belgium’s framework is seen as a potential model for other European countries seeking to adopt phage therapy. While the magistral preparation approach is proving useful, there is a need for more randomized controlled trials to further evaluate phage therapy’s effectiveness and to explore potential synergies with antibiotics [19].

PHAGOBURN was a European Research & Development (R&D) project funded by the European Commission under the 7th Framework Programme for Research and Development. This clinical trial aimed to assess the safety and efficacy of topical bacteriophage therapy for *Escherichia coli* (*E. coli*) or *Pseudomonas aeruginosa* (*P. aeruginosa*) wound infections in burn patients. The trial was registered with the European Clinical Trials database (EudraCT number: 2014-000714-65) and ClinicalTrials.gov (NCT02116010), and it has now been completed [20].

Since their discovery, the exploitation of bacteriophages for therapeutic purposes has taken place; phages were successfully applied against intestinal infections [21,22]. Then, the commercial production of phage preparations directed against several bacterial infections such as plague and cholera was triggered [22,23].

However, with the discovery of antibiotics in the 1940s, phage therapy was abandoned all over the world except in the countries of the former Soviet Union [23,24]. Thus, phage therapy has faced many difficulties, namely the insufficient understanding of fundamental biological basic notions about phages, the difficulty of having pure phage preparations without bacterial debris, restricted lytic spectra, problems of standardization and patenting, and the advent of antibiotics [25,26,27].

Nevertheless, we are currently experiencing a different context, namely the emergence and spread of resistance to antibiotics. The emergence of bacteria resistant to one or more antibiotics represents a major global health threat. In this context, scientific research is increasingly oriented towards phage exploitations, focusing on the discovery of new bacteriophages with antimicrobial activity to fight bacterial infections (Figure 1).

This review focuses on the historical aspects of research on phages, namely their applications related to the resolution of human problems, from health to environmental problems, trying to discern what role they will play in the future dynamics of human beings.

## 2. Bacteriophages

### 2.1. Suitability of Phages for Different Applications

Bacteriophages are obligate intracellular parasites. To multiply, they require a bacterial host cell. There are 2 types of infectious cycles in phages. There are virulent or so-called lytic phages which follow a lytic cycle which ends with the lysis of the host and the release of new assembled virions. On the other hand, there are temperate phages which carry out a lysogenic cycle.

The lysogenic cycle begins when temperate phages insert their genetic material into the bacterial chromosome, forming a prophage. In this state, the phage DNA replicates passively along with the host genome and is inherited by daughter cells, but no new infectious phage particles are produced. The prophage can remain dormant for extended periods until specific conditions, such as environmental stress or changes in the metabolic state of the host, trigger its induction into the lytic cycle. In contrast, lytic phages exclusively follow the lytic pathway, immediately producing new virions and lysing the host cell. The use of phages in therapy favors lytic phages over temperate phages because temperate phages carry a risk of transferring virulence factors [10,28].

While both lysogenic conversion and superinfection immunity are relevant factors in phage therapy, the primary reason lytic bacteriophages are favored over temperate phages is superinfection immunity. Temperate phages, which can enter a lysogenic cycle, can establish lysogeny where the phage genome integrates into the host bacterium’s DNA, making the bacteria immune to further infection by the same type of phage. This immunity can prevent the phage from effectively clearing an infection, as it can no longer infect and kill the already-lysogenized bacteria [29]. The latter involves several steps, starting with the step of adsorption, followed by the transfer of DNA into the host cell, and the replication of phage DNA step, the next step is the transcription and translation of phage DNA, encapsidation of the latter, the assembly of phage particles and eventually the release of the newly assembled virions.

The fields of exploitation of bacteriophages are diversified, for example, in molecular biology, phage therapy, detection of bacteria, control and prevention of bacterial contamination of foodstuffs, and decontamination of wastewater. Nevertheless, important selection criteria of a phage for such exploitation deserve attention:

**A strictly lytic phage:** The phage used must be lytic, in other words incapable of integrating its genome into that of its host strain, which promotes gene transfer. Indeed, temperate phages can be used by bacteria as transduction vectors, i.e., to transfer virulence genes from one host to another [30]. Therefore, the use of non-pathogenic host strains is more secure for the amplification and propagation of bacteriophages [30,31].

**The absence of virulence factors in the phage genome:** Phages that harbor virulence genes in their genomes constitute a risk for human health. It is not recommended for use in medical and food applications. Sequencing the phage genome is a mandatory step to ensure their safety, since some *Enterobacteriaceae* phages are capable of synthesizing toxins [31,32].

**The long-term stability of the phage:** The stability of a phage vis-à-vis certain external factors (temperature, salinity, pH, chemical detergents, etc.) is an important criterion for industrial preparations and applications of phages [33]. The long-term viability of the phage and the maintenance of a high concentration during storage give an idea of its therapeutic potential. Freeze-drying [34] and micro-encapsulation [35] are two techniques that improve phage stability.

**A wide lytic spectrum:** Bacteriophages are very specific and restricted even to one species and to certain strains of a species. However, significant versatility has been detected in *Enterobacteriaceae* phages [36]. Phages with versatile lytic spectra are considered potential antimicrobial agents and good biocontrol candidates [37].

It is important to use a polyvalent phage meeting all of these selection criteria, but the exploitation of phage cocktails remains the most effective method for obtaining more promising results by aiming for a more abundant bacterial coverage. Cocktails usually also prevent or delay the emergence of phage-resistant bacteria [3,38].

### 2.2. Legislation for Bacteriophage Preparations

The regulation of bacteriophages is still evolving and may not be as well-established as that of traditional antimicrobial agents in both Europe and the United States. While there are regulations in place for certain applications, such as food safety, the regulatory framework for other specific uses of bacteriophages, like phage therapy, is still developing. The application of bacteriophage during food packaging, it is an evolving area, and different countries have different laws and regulations in place. However, in general, phage-based food packaging is regulated by food safety agencies, such as the United States Food and Drug Administration (US) (FDA) and the European Food Safety Authority (EFSA). In the US, the FDA considers phages to be a form of food additive, and they must be reviewed and approved by the agency before they can be used in food packaging. This process typically involves submitting data on the safety, efficacy, and stability of the phages, as well as data on the materials used in the packaging. In the European Union, the use of phages in food packaging is governed by regulation (EC) No. 1935/2004 on materials and articles intended to come into contact with food, and regulation (EU) No. 528/2012 on biocidal products. These regulations outline the requirements for the safety and efficacy of food contact materials, including phage-based food packaging. Overall, the regulations for bacteriophages are evolving, and there may be ongoing discussions and efforts to establish more comprehensive guidelines and frameworks for their use. Perhaps the biggest obstacle to the implementation of phage therapy in Western medicine is the lack of proper regulation. If phage preparations are considered “conventional” drugs, they must comply with the corresponding legislation regarding the production and quality of drugs. This basically means that they have to follow good manufacturing practice requirements, which has posed a significant problem for the development of medicinal phage preparations in terms of increased cost or management complexity to develop a large-scale production [39]. Although phage therapy is truly considered a drug, the nature itself of phage makes it absolutely different from current antimicrobial chemotherapy. Indeed, phage-host co-evolution, bacteriophage specificity or complex pharmacological behavior in vivo have negatively influenced the outcome of many phages’ clinical trials [20,40].

Therefore, the path from clinical trials to market still seems quite distant. A provisional conclusion to be accepted is that the evaluation of phage therapy will be based on specific adapted parameters and may be different from that of chemotherapies [40,41]. To date, the most reliable approach to clinical phage therapy is the use of tailored phage preparations designed to target the specific bacterial infection in an individual patient. Indeed, the compassionate use of patient-specific phages, particularly when no better treatment options are available, represents the current regulatory framework for phage therapy in most countries [42,43]. Within this framework, some of the more stringent requirements can be circumvented, even though it still requires the approval of the competent authorities in each particular case. A strategy recently implemented in Belgium has enabled a more systematic and flexible approach to personalized phage therapy. This method utilizes master phage preparations, which, unlike conventional drugs, are subject to less stringent regulations regarding production and marketing [19,44]. In this context, phages are regarded as active pharmaceutical ingredients. A practical infrastructure to operate under this regulatory framework would be a dedicated phage bank in which each conserved phage should be certified to be used as an active pharmaceutical ingredient, with the certification ensuring all essential quality attributes are met, including a “genetic passport” for each individual phage [19,45].

## 3. Bacteriophage Applications

### 3.1. Bacteriophages and Molecular Biology

Bacteriophages are considered among the simplest systems in comparison with a cell. The ease of their manipulation and production have qualified it as a model of choice for the study of living mechanisms. Indeed, researchers Max Delbrück, Alfred Hershey and Salvador Luria have made major discoveries in the field of molecular biology. Among them, the phenomenon of recombination was highlighted at the end of the 1940s: the co-infection of a bacterium by two different phages gave rise to hybrid phages, and phages damaged by radiation could be “repaired” through genetic exchange with other phages infecting the same bacterium [46]. Series of works spanning from 1928 to 1952 will make it possible to definitively associate DNA with the notion of genetic information. Hershey and Chase used a technique that aims to use radioactive isotopes as markers, making it possible to follow the fate of macromolecules. The results of this work demonstrated that only phage DNA enters the host cell and that it is enough to trigger the production of new phages, this experiment was known as the Hershey-Chase experiment [17]. Thus, studies carried out on phages have shown the role of RNA as an intermediary between DNA and proteins [47,48]. The restriction/modification system was a defense process adopted by bacteria against bacteriophages. This mechanism was discovered in the 1960s. Arber discovered restriction enzymes that degrade DNA. A means of defense adopted by the bacterium against any foreign DNA. In addition, the modification system allows the DNA to be protected from restriction enzymes. The cited results were confirmed by Smith’s discovery. He was able to purify a restriction enzyme and demonstrate that it cuts DNA in the middle of a specific symmetrical sequence. Finally, Nathans was the first to consider the possible applications of this system, in particular to establish genetic maps [49]. Similarly, other systems derived from bacteriophages are widely used in molecular biology. Phage T4 DNA ligase is used to ligate between segmented DNA molecules. The restriction enzyme + DNA ligase system was a very practical and still commonly used tool for manipulating DNA. Lambda phage was developed as a cloning vector. The T7 phage DNA polymerase was used for sequencing, and the T7 RNA polymerase was used for the expression of genes under the control of the T7 promoter [50]. The Clustered Regularly Interspaced Short Palindromic Repeats Associated protein Cas system (CRISPR-Cas system) discovered in 2007, confers adaptive immunity to bacteria against all foreign DNA including phage DNA [50,51]. In 2021, Locus Biosciences conducted the first clinical trial using a CRISPR-enhanced bacteriophage therapy. This trial involved using (CRISPR-Cas3) to improve the ability of bacteriophages to kill *E. coli* bacteria, specifically targeting urinary tract infections. The trial demonstrated the safety and efficacy of this approach, with promising results in reducing bacterial levels in patients [52].

The contributions of the study of phages to modern molecular biology are very important and are used daily without us sometimes remembering the origin of these techniques, whether they are in vitro transcription systems using T7 bacteriophage RNA polymerase or site-specific recombination cloning systems such as Crelox or C31, which allow for high-throughput, scar-free cloning [53].

Currently, one of the latest high-throughput sequencing technologies (PAC Bio) uses the DNA polymerase properties of bacteriophage phi29. Specifically, PacBio sequencing utilizes phi29 DNA polymerase for its speed, processivity, efficiency, and fidelity during DNA replication. In particular, the spectacular applications of anti-bacteriophage system (better known as CRISPR-Cas9), prove that bacteriophages and their interactions with CRISPR-Cas systems will continue to be major players in future developments [53].

### 3.2. Phage Therapy

Historically, there has been an accelerated transition of phage therapy from the bench (theoretical) to the clinic (practical).

Phage therapy is the application of specific viruses with the aim of reducing or eliminating pathogenic or harmful bacteria. It is a bactericidal alternative that aims to use natural bacteriophages as antimicrobial agents. Nowadays, there is renewed interest in the use of phages in medicine mainly due to the emergence of multi-antibiotic-resistant pathogenic bacteria [38]. Phages are characterized by their ease of manipulation and production. Indeed, bacteriophages replicate exponentially in the presence of their hosts. Phages can be administered by multiple routes (oral, intravenous, intraperitoneal, topical or intranasal) [54].

The beginnings of the golden age of phage therapy were marked by the work of Félix d’Hérelle. After obtaining promising results on animals in 1918, he treated young children with bacillary dysentery caused by *Shigella* at the Necker Hospital in Paris in 1919. Subsequently, the positive results found by Félix d’Hérelle have been published in his book “The bacteriophage: its role in immunity” [55,56]. The marketing of phage-based preparations was started in 1928 in France by the Robert and Carrier laboratories. During the 1930s, the ten best-selling medicines were reserved for the five phage-based specialties (bacté-intesti-phage; bacté-coli-phage; bacté-rhino-phage; bacté-pyo-phage; bacté-staphy- phage) [5,57].

In 2001, phage therapy regained interest because of the growing problem of antibiotic resistance. While the initial discovery and early therapeutic applications of phages occurred in the early 20th century, the rise of antibiotics in the mid-20th century largely overshadowed phage therapy. However, by the early 21st century, the limitations of antibiotics led scientists to re-evaluate phage therapy as a potential alternative or adjunct treatment [58]. The effectiveness of phage therapy in treating infections caused by Enterobacteriaceae was later reinforced by clinical trials carried out in veterinary medicine. Phages are able to reduce embryo mortality in chickens infected with an avian pathogenic *E. coli* strain [59]. Phage cocktails have been applied to control and eliminate intestinal infections caused by *E. coli* O157:H7 in ruminants [60]. Another phage cocktail has been used to treat diarrhea caused by strains of *E. coli* in piglets, lambs and calves [61,62].

Several studies of phage therapy to destroy *Enterobacteriaceae* have been done on mice; [63] demonstrated, through an in vivo study, the therapeutic potential of a phage to rescue a group of mice infected with an extended-spectrum beta-lactamase (ESBL)-producing *E. coli* strain. Oral administration of an *E. coli*/*Salmonella* spp./*Listeria monocytogenes*-targeting bacteriophage cocktail (tentatively named F.O.P) was effective in treating mice infected with *E. coli* O157:H7 [64]. The efficacy of the myPSH1131 phage has been shown both in vitro and in vivo to reduce *E. coli* [65]. The efficacy of SS phage in the treatment of *Klebsiella pneumoniae*-mediated lobar pneumonia in mice has been proven in vivo [66]. Another study showed the therapeutic potential of phages to significantly improve the protection of mice with respiratory infections caused by *K. pneumoniae* [33].

All of the works cited have shown promising results for phage therapy, making this natural antimicrobial a renewed interest in the exploitation of phages in medicine to help us prevent or treat diseases caused by Enterobacteriaceae. This evaluation was carried out orally on 15 healthy volunteers in Switzerland showing the safety of oral administration of phages [67]. Another study took place in Bangladesh, also tested the level of safety of a commercial phage cocktail that is prepared by a Russian company. This phage preparation, comprising different groups of phages, mainly T4viruses and T7viruses, is intended for oral application on healthy human subjects [68].

From the end of the 1980s, phages were adapted for biotechnological applications and in nano-medicine [69,70]. Among these applications is the “phage display” (Figure 1). This technique, introduced in 1985 by G. P. Smith, is widely used today [69]. It allows a protein of interest to be exposed to the surface of a phage by fusing its gene to that of a capsid protein. Phage display thus allows for the screening of protein libraries and the selection of those that exhibit a desired function (enzymatic activity, high affinity for a ligand, etc.). Phage capsids are of renewed interest due to their robustness, their capacity for self-assembly and their homogeneity. Thus, empty DNA capsids called “virus-like particles” (VLPs) can be used in multiple applications. In particular, VLPs can be used as antigen presenters, or used in vaccination. Particle addressing is a therapeutic tool of interest; a VLP is internalized in a capsid or attached to its surface and it can be directed towards a particular tissue thanks to a function making it possible to target certain cells. VLPs can also be used in imaging, as contrast agents to perfectly visualize different tissues. To increase the rate of sensitivity and specificity, contrast agents can be linked to capsids to detect particular tissues [69].

However, immunological responses of phages can vary, particularly depending on prior infection with host bacterial strains, and with different phage strains [70]. In recent years, it has been recognized that bacteriophages have been proposed as delivery vehicles for protein- and DNA-based vaccines for coronavirus disease of 2019 (COVID-19) [71]. They are also used as delivery vehicles in gene therapy and as tools for screening proteins, peptide or antibody libraries [25,72]. A study took place in Pennsylvania to study the cytogenetic, proliferative and viability effects of 4 bacteriophages on human lymphocytes. The results showed that the administration of these 4 phages delayed the synthesis of bacterial DNA, damaged the membrane of *E. coli* and affected DNA, RNA and protein synthesis. Vaccine use does not affect normal lymphocyte kinetics in recipients infected with *E. coli* [73]. The immune response following the production of antibodies through the administration of phages affected their survival in the intestine, but there is still treatment efficacy [74]. The immunogenic property developed by bacteriophages has led to investigations of their use in vaccinations, serving as transfer vectors of DNA incorporated into their genomes or peptides displayed on their surfaces, triggering a high and long-lasting antibody response [75]. However, from a therapeutic point of view, phage derivatives are more favored to circumvent the immune response [76]. It is for this reason that the exploitation of the enzymatic activity of phage derivatives, in particular endolysin, has been suggested to be used to degrade the peptidoglycan layer of the host bacterial cell [77,78].

Among the newly developed strategies, Phage-Antibiotic Synergy: bacteriophages can also serve as promising adjuncts to antibiotics for the treatment of bacterial infections. Although the multiplication of phages has a substantial impact on the biosphere, the external environment of which affects the production of phages. Researchers have shown that non-lethal concentrations of certain antibiotics can significantly stimulate the production by the host bacterial cell of certain virulent phages. A low dose of cefotaxime, a cephalosporin, increased the production of wMFP phage by the uropathogenic *E. coli* strain more than 7-fold. A related effect was observed in various host-phage systems, including T4-like phages, with beta-lactam and quinolone antibiotics, as well as mitomycin C [79]. Another similar study showed that phage treatment with progressively higher concentrations of antibiotics (meropenem, ciprofloxacin and tetracycline), resulted in increased plaque size and phage titers. The results suggest that antibiotics can be combined with phages to stimulate increased production and or activity of phages and thus improve the efficiency of bacterial destruction [80]. A study carried out in France has shown that the combination of antibiotics (ciprofloxacin) and phages effectively reduces the growth of *E. coli* and considerably limits the emergence of cells resistant to antibiotics and phages. Thus, this combination decreased the number of living cells compared to phages used alone and antibiotics alone [37].

Currently, the increasing prevalence of infections caused by multidrug-resistant bacteria is worrying. It has thus become urgent to develop new anti-infective strategies. Though the use of phages as antibacterial agents is not a universal solution, it is realistic both in economic and temporal terms. In the light of the fundamental knowledge obtained during more than 100 years of research, rigorous medical and scientific approaches are now being put in place to remove the last obstacles to the reintroduction of phage therapy in human medicine in countries that have forgotten or neglected this therapeutic approach [53]. Since the year 2000, over 100 English-language publications detail human phage applications, including case studies and clinical trials. These studies explore the use of bacteriophages to target and kill specific bacteria, both in traditional phage therapy and in novel applications [81]. PhagoDAIR I trial has received authorization from the French National Agency for the Safety of Medicines and Health Products (ANSM) in 2022, highlighting its significance (NCT05369104). This trial was conducted by Phaxiam Therapeutics and aimed to evaluate bacteriophages as an alternative to antibiotics for treating resistant *S. aureus* infections. The PhagoDAIR I study found a 74% infection control rate in patients with osteoarticular infections caused by *S. aureus* following a single intra-articular phage injection. Phaxiam received approval from the US FDA in November 2024 for a Phase II trial, aimed at proving the clinical efficacy of phages against multidrug-resistant bacterial infections. Some examples of clinical trials are included in Table 1.

There are currently several clinical trials concerning phage therapy of *Staphylococcus aureus* (*S. aureus*), *P. aeruginosa*, *E. coli* infections, and other bacterial pathogens in the Clinical Trials database (ClinicalTrials.gov Identifiers NCT05269134, NCT05010577, NCT03808103, NCT05537519, NCT05269121, NCT04650607, NCT05269134, NCT04323475, NCT05177107, NCT04682964, NCT05369104, NCT05314426, NCT04724603, NCT05272579, NCT05618418, NCT05590195, NCT04325685, NCT03326947, NCT05184764, NCT05488340, NCT04791644, NCT04708704, NCT02664740, NCT05453578, NL-OMON53569, …). However, these studies are still in their early phases and there are no reported results available at the time of writing. But on the other hand, some studies have been carried out but no results have been published.

Here, we present some results of clinical trials. Two T4 coliphage preparations were randomized in Bangladeshi children hospitalized for acute bacterial diarrhea. This study was funded by a grant from Nestlé Nutrition and Nestlé Health Science. The clinical trial was registered under the identifier NCT00937274 on ClinicalTrials.gov. The safety of oral phage therapy was assessed clinically and by functional assays; coliphage and *E. coli* titers, as well as enteropathogenesis, were determined in stool, and quantitative diarrhea parameters were measured. The fecal microbiota was studied by 16S rRNA gene sequencing; the genomes of four fecal Streptococcus isolates were sequenced. Oral coliphages have shown safe intestinal transit in children but no intestinal amplification or improvement in diarrheal outcome, probably due to insufficient phage coverage and/or low phage titers, requiring higher oral phage doses. For successful phage therapy, it is necessary to acquire more in-depth knowledge on phage–bacteria interaction in vivo and the role of *E. coli* in childhood diarrhea [82].

**Table 1:** Recent clinical trials/studies of potential phage therapies.

Clinical Trial/Study	Outcomes	Country	Reference
A Human Experimental Model to Evaluate the Safety and Efficacy of the ShigActive™ Bacteriophage Preparation for Shigellosis	ShigActive™, a bacteriophage preparation, is being evaluated in a clinical trial to assess its safety and efficacy for treating shigellosis.	USA	NCT05182749
Assessing the Safety, Tolerability, and Pharmacokinetics of LBP-EC01 in Patients with *E. coli* Colonization of the Lower Urinary Tract	No drug-related treatment-emergent adverse events (TEAEs) were observed in this Phase 1b study, confirming that LBP-EC01 is safe and well tolerated. The clear pharmacodynamic difference between LBP-EC01 and placebo, regardless of MDR status, supports its potential for treating antibiotic-resistant *E. coli* urinary tract infections.	USA	NCT04191148
Bacteriophage PhiX174-based assay of in vivo immune competence in patients with HIV	Immunization with bacteriophage PhiX174 revealed persistent abnormalities in CD4(+) T-cell function in most patients with HIV, even after adequate antiretroviral therapy.	USA	NCT00001540
Bacteriophage-Based Treatment for Urinary Tract Infections in Patients Undergoing Transurethral Resection of the Prostate	Although efficacy was similar to placebo, this trial provides valuable insights for designing future large-scale studies on phage therapy for urinary tract infections.	Georgia	NCT03140085
Beta Testing of *S. aureus*/MSSA/MRSA Blood Culture with Microphage Agent	Microphage is developing innovative phage-based immunoassay solutions for rapid bacterial identification and antibiotic susceptibility testing.	USA	NCT00814151
Efficacy of T4N5 Liposomal Lotion in Preventing Nonmelanoma Skin Cancer Recurrence in Kidney Transplant Patients	T4N5 liposomal lotion has shown promise in reducing nonmelanoma skin cancer recurrence among kidney transplant recipients.	USA	NCT00089180
Evaluating the Safety, Tolerability, and Fecal Pharmacokinetics of BX002-A in Healthy Adults: A Phase 1 Study	BX002-A was safe and well tolerated in healthy adults. Pharmacokinetic analysis revealed high levels of viable phages in stool, confirming interaction with target bacteria after gastrointestinal passage.	USA	NCT04737876
Evaluation of Changes in Inflammatory Markers Following Treatment with Bacterial Viruses	This case study showed that a single bacteriophage can effectively resolve a bacterial infection.	Poland	[83]
Evaluation of the bacteriophage cocktail TP-102 for treating diabetic foot ulcers	A preliminary safety assessment revealed no serious adverse events related to the preparation. Final efficacy results are pending.	Israel	NCT04803708
In subjects with non-cystic fibrosis bronchiectasis and chronic pulmonary *P. aeruginosa* infection, the safety, phage kinetics, and efficacy of inhaled AP-PA02 were evaluated (Tailwind).	A Phase II clinical trial, named Tailwind, is currently recruiting to evaluate the safety, efficacy, and kinetics of AP-PA02 in patients with non-CF bronchiectasis and chronic *P. aeruginosa* infections.	USA	NCT05616221
Intravenous and Nebulized Bacteriophage Therapy for Multidrug-Resistant Acinetobacter baumannii Respiratory Infection	Successful treatment led to favorable clinical response and bacterial eradication.	USA	[84]
Phage Therapy for Multidrug-Resistant Burkholderia multivorans Infection in a Cystic Fibrosis and Lung Transplant Patient	Phage therapy was followed by transient clinical improvement and decreased bacterial DNA in respiratory samples without seroneutralization.	USA	[85]
PhagoDAIR I: Pilot Study of Phage Therapy for Osteoarticular Infections on Prostheses (Targeting *S. aureus*).	The PhagoDAIR I study demonstrated excellent safety for PHAXIAM’s phages, with a 74% infection control rate after a single intra-articular injection. These results support the Phase II GLORIA study.	France	NCT05369104
Pregnane X Receptor (PXR) as a Regulator of Intestinal Permeability and Indole Signaling in Inflammatory Bowel Disease	The study emphasizes the need for both in vitro and in vivo analyses to fully understand the therapeutic utility of PXR-directed analogs.	USA	NCT04089501
Prospective Evaluation of Direct-from-Positive Blood Culture Performance of the Microphage *S. aureus*/MSSA Test	The KeyPath MRSA/MSSA test achieved 91.8% sensitivity and 98.3% specificity for MRSA/MSSA detection, providing faster and more effective results than traditional methods.	Not specified	NCT01184339
Prospective, Randomized, Double-Blind, Controlled Study of WPP-201 for Venous Leg Ulcers	The product was deemed safe; a Phase II study is planned to evaluate its efficacy.	USA	NCT00663091
Safety and Immunogenicity of a Recombinant Human CD40 Ligand in Patients with X-Linked Hyper-IgM Syndrome	The findings suggest potential improvements in dendritic cell activation and tumor immunity, with possible applications for cancer vaccines.	USA	NCT00001145
Safety, Recovery, and Pharmacodynamics of Multiple Oral Administrations of SNIPR001 in Healthy Subjects	SNIPR001 demonstrated safety and efficacy in Phase 1, supporting further development of an intravenous formulation for preventing bloodstream infections in cancer patients.	USA	NCT05277350
Therapeutic Efficacy and Safety Profile of the Bacteriophage Preparation Biophage-PA in Antibiotic-Resistant *P. aeruginosa* Chronic Otitis.	A double-blind, placebo-controlled Phase I/II trial demonstrated that phage treatment was safe and improved symptoms.	United Kingdom	[86]
Treatment of Multidrug-Resistant *P. aeruginosa* in Cystic Fibrosis with Personalized Inhaled Bacteriophage Therapy	Phage therapy reduced bacterial density in sputum and improved lung function, with no serious adverse effects.	USA	[87]

The first Food and Drug Administration (FDA)-approved clinical trial in the US for intravenous bacteriophage therapy was approved in 2019 and was conducted by researchers at the University of California San Diego. This trial evaluated the safety, tolerability, and efficacy of an experimental bacteriophage therapy for patients with *S. aureus* infections related to ventricular assist devices. The therapy was administered intravenously and used in conjunction with antibiotic treatment. In essence, this trial was a groundbreaking step in exploring the potential of phage therapy as a viable treatment option for severe bacterial infections, particularly those resistant to traditional antibiotics [88].

A recent study by [87] showed promising results, in which treatment with personalized lytic phages administered via nebulization led to a reduction in sputum bacterial load and an improvement in lung function. The therapy was well tolerated and resulted in decreased bacterial antibiotic resistance and virulence, reflecting an evolutionary trade-off associated with the development of phage resistance.

An American team reported the successful treatment of a patient in intensive care with a severe respiratory infection caused by multidrug-resistant Acinetobacter baumannii. Following the failure of conventional treatments, a personalized phage-based therapy—administered both intravenously and via nebulization—was applied to target the infection at different levels (systemic and local, in the lungs). This combined approach led to significant clinical improvement and eradication of the infection, without notable adverse effects. This case highlights the potential of phages as an alternative or complement to antibiotics in the management of severe and resistant nosocomial infections, particularly in intensive care settings [84].

On the other hand, in another study, a lung transplant patient with cystic fibrosis and *Burkholderia multivorans* infection received inhaled phage therapy for seven days prior to her death. Although the outcome represented a clinical failure, this case highlights the current limitations, uncertainties, and challenges associated with phage therapy for resistant infections [85].

In 2019, genetically modified bacteriophages were used for the first time in human therapy to treat a multidrug-resistant *Mycobacterium abscessus* (*M. abscessus*) infection in a 15-year-old cystic fibrosis patient. The patient received a three-phage cocktail, with two of the phages being genetically engineered to enhance their lytic activity. This marked a significant milestone in the field of phage therapy, demonstrating the potential of engineered phages to combat antibiotic-resistant bacteria [89,90]. These recent advancements in phage engineering are poised to revolutionize future phage therapy by enhancing efficacy, precision, and adaptability in combating antibiotic-resistant infections. Innovative strategies include genetic modifications to extend host range and improve stability, as well as chemical modifications to enhance phage delivery and resistance to environmental factors. Moreover, the development of synthetic phages through DNA synthesis allows for the design of phages with tailored properties, such as increased stability and targeted delivery, offering a promising approach to personalized medicine [91].

#### Phage Therapy in the Context of Antimicrobial Stewardship

Antimicrobial stewardship (AMS) programs are designed to optimize antimicrobial use, improve patient outcomes, and impede the spread of antimicrobial-resistant organisms (AMR) [92]. In that respect, bacteriophage therapy aligns well with AMS goals. Phages can selectively target pathogenic bacteria while sparing the commensal microbiota, thereby reducing the collateral selective pressure that broad-spectrum antibiotics impose [72]. Moreover, phage-antibiotic combination treatments, where phages are used alongside antibiotics, have been shown to enhance bacterial clearance and limit resistance emergence, thus extending the lifespan of existing antimicrobials [37]. On the other hand, from a one-health perspective [93], integrating phage interventions into AMS frameworks can support both human and environmental health: phages can reduce antibiotic usage in clinical care and may help prevent the dissemination of resistance genes through wastewater, agriculture and food-production systems.

In this context, to explore their potential, phage therapies must be supported by surveillance systems, standardized manufacturing and regulatory pathways, and stewardship policies that include phage use in antibiotherapy protocols. Overall, by reducing antibiotic exposure, targeting pathogens more precisely and integrating into cross-sector stewardship efforts, phage therapy could represent an important asset to AMS strategies, as well as an important tool in the battle against AMR.

### 3.3. Detection of Bacteria

Bacteriophages, or phages, are indeed known for their high specificity in targeting bacterial strains, which makes them useful for rapid and efficient bacterial detection. This specificity stems from the phage’s ability to only bind and infect specific bacteria, leading to targeted detection. Several detection methods have been considered. One of these methods uses specific labeled antibodies to bacteria-bound phages [94,95,96,97]. A second strategy which is characterized by its simplicity and rapidity consists of applying lysates of phages with broad lytic spectra within the same species directly on an inoculated bacterial mat with an unknown strain to monitor the appearance of lysis plaques. It allows for the detection of strains of *E. coli* O157:H7 [98].

This detection method was used as a preliminary tool for rapid identification and can be complemented by the technique of pulsed field electrophoresis for more specificity. Therefore, phage typing and pulsed Field Gel Electrophoresis (PFGE) fingerprinting represent complementary procedures for subtyping serogroup O157 of *E. coli* and that the combined use of these procedures provides optimal discrimination [99,100]. In 2001, a research team first demonstrated the successful use of a phage lysin to prevent and eliminate a bacterial infection in a living organism. This breakthrough involved the use of a recombinantly produced and purified lysin to target and lyse Gram-positive bacteria [101].

Rapid and early detection of foodborne pathogens is central to the food industry to maintain safe food free of pathogens. Thus, the pursuit of novel biological recognition systems for detecting pathogens in food and production environments remains an active area of research. Although traditional bacteriological techniques provide high accuracy, they are often labor-intensive and time-consuming. Moreover, these methods are generally unsuitable for on-site applications because they require complex sample preparation and lengthy incubation steps. Advances in phage-based biosensors for the detection of foodborne pathogens have recently been reviewed by [102].

### 3.4. Phage Remediation

#### 3.4.1. Control and Prevention of Foodstuff Contamination

In 2006, the US FDA approved the use of a bacteriophage preparation, specifically LMP-102, as a food additive for ready-to-eat meat and poultry products. This marked the first time the FDA approved the use of a phage preparation to be added to food products [103]. For example, *Enterobacteriaceae* are responsible for several foodborne diseases. Indeed, human food, either vegetable (vegetables, fruits, mushrooms) or animal (dairy products, fish, meat) is very heterogeneous and a potential risk factor for public health. Food products have often been debated as a vector for the transfer of bacteria. Food contamination can occur throughout the production chain and even during transport [104,105].

The development of new methods to control microbial contamination in food and the food processing environment is extremely important. The use of phages as food additives to assess the potential of bacteriophages in the control and decontamination of foodstuffs is of paramount importance for public health. In this context, several studies have been carried out. A US study has shown that phages are promising as a potentially viable strategy to reduce *Salmonella* in pigs before slaughter [106]. Another similar study showed that phage cocktails (ΦSH17, ΦSH18, and ΦSH19) could be used post-slaughter as a means to reduce *S. typhimurium* contamination of pig carcasses [107]. Anti-*Salmonella* phage cocktail significantly reduced *Salmonella* levels. Thus, additional in vitro studies showed that this phage cocktail was also lytic against several non-*Typhimurium* serotypes [108]. Virulent phage cocktails were able to significantly reduce *Salmonella* genus contamination in broiler chickens [3].

The main route of transmission of *E. coli* O157:H7 in humans is through foods of bovine origin. Numerous studies have been carried out in vivo with the aim of controlling the colonization of *E. coli* O157:H7 in cattle [109]. Ref. [110] assessed the effectiveness of oral and rectal administration of bacteriophages specific to *E. coli* O157 with the aim of reducing fecal excretion of this germ in experimentally inoculated cattle. The study aimed to determine if bacteriophages could be used as a biocontrol agent to reduce the presence of these harmful bacteria in cattle feces. The study involved experimentally inoculating steers with *E. coli* O157:H7 and then administering bacteriophages via different routes (oral and rectal). The researchers monitored fecal shedding of the bacteria over time to assess the impact of the phage therapy. The results indicated that while both oral and rectal administration of bacteriophages led to a reduction in fecal *E. coli* O157:H7 shedding, the effectiveness varied. The study found that the overall mean fecal shedding was higher in steers treated rectally compared to those treated orally. Additionally, some steers in both treatment groups continued to shed the bacteria, suggesting that phage therapy alone may not completely eliminate *E. coli* O157:H7 in all animals. The study also highlighted the importance of considering the risk for bacterial resistance to phages and the need for further research to optimize phage therapy strategies for *E. coli* O157:H7 control in cattle.

Trials were conducted on bacteriophages in an effort to reduce *E. coli* O157:H7 naturally residing in the intestines of sheep. The findings showed that treatment with a combination of two phages, CEV1 and CEV2, led to a significant reduction in bacterial concentrations within the cecal and rectal contents. Reference [111] evaluated the use of a three-phage mixture to reduce *E. coli* O157:H7 on beef. Phage immobilization on a modified cellulose membrane has been shown to be effective in targeting *L. monocytogenes* and *E. coli* O1576:H7 in raw meat [112].

The biological control potential of bacteriophages against *Salmonella* was examined on fresh cut fruit [113]. Comparable studies have assessed the antimicrobial effectiveness of bacteriophage mixtures, both individually and in combination with trans-cinnamaldehyde essential oil, against various *E. coli* O157:H7 strains contaminating lettuce and spinach leaves [114]. Their results showed that using the phage or the oil individually successfully inhibited the growth of *E. coli* O157:H7 on both leaves after 24 h. However, when the two treatments were combined, no survivors of bacteria were detected. These results indicated that the combination of phage and the essential oil of trans-cinnamaldehyde was highly effective against enterohemorrhagic *E. coli* on both leafy greens. This combination could potentially be used as an antimicrobial to inactivate enterohemorrhagic *E. coli* O157:H7 and reduce their incidence in the food chain. Numerous foodborne outbreaks have been associated with processed food products such as ready-to-eat meals, cheeses, milk, and powdered infant formulas.

The work of [115] evaluated the bio-therapeutic capacity of a single bacteriophage SJ2 against *Salmonella enteritidis* (*S. enteritidis*) during the manufacture, ripening and storage of cheddar cheese made from raw and pasteurized milk. The findings reported in [115] indicated that after 89 days of storage at 8 °C, *Salmonella* was no longer detectable in cheese produced from pasteurized milk. In a similar study, ref. [116] evaluated the efficacy of phage F01-E2 in reducing *S. typhimurium* in a variety of ready-to-eat foods. Phage-treated foods resulted in complete eradication of *Salmonella*.

Although research on the use of phage-derived enzymes in food applications is more limited compared to their use in human and veterinary medicine, their potential as biological control agents within the food industry is gaining growing recognition [117,118,119]. Endolysins are peptidoglycan-degrading enzymes produced by bacteriophages at the terminal stage of their lytic life cycle [120,121]. They are particularly effective when applied exogenously to Gram-positive bacteria, resulting in immediate bacteriolysis. In the field of biological control of *Salmonella*, ref. [122] conducted studies with a P22 phage endoglycosidase (P22sTsp) targeting *S. typhimurium*. Oral administration of P22sTsp to chickens infected with *S. typhimurium* resulted in a significant reduction in the pathogen in the intestine and prevented re-entry into internal organs. The authors proposed that the resistance of these spiked proteins to acid-induced denaturation and proteolytic digestion within the gastrointestinal tract makes them promising candidates for oral administration against Gram-negative bacterial pathogens in animal feed.

Food preservation has always been a necessary part of food production. Regardless of current preservation techniques and the fact that increased research has led to a better understanding of how microbial food spoilage occurs, large amounts of food produced globally each year are lost to microbial spoilage [123,124,125]. Concerning fruits and vegetables, ref. [126] demonstrated in field experiments the successful use of phages to control bacterial spot of tomato. Application of six different species-specific phages significantly reduced the severity of bacterial spotting in contaminated tomatoes. The authors found that phage-treated fruit resulted in an increase in the amount of marketable product compared to non-treated control fruit.

Biofilm formation has been widely observed in various industrial settings. These structures act as a protective growth mechanism, enabling bacteria to withstand and persist under unfavorable environmental conditions [127,128]. Ref. [129] demonstrated the ability of bacteriophage T4D+ to disrupt, infect and multiply in the exopolymer matrix of an *E. coli* 3000 XIII on the polyvinyl chloride surfaces.

This finding, coupled with the increasing prevalence of antibiotic-resistant bacteria, the decline in pharmaceutical research for new antibiotics, consumer demands for pathogen-free food, and the antimicrobial properties of phages, has spurred interest in phage-based products.

Diminishing antibiotic efficacy, the strong decrease in the number of pharmaceutical companies investing in new drugs under development, consumer demands for the production of pathogen-free foods, and synthetic chemicals associated with undeniable antimicrobial properties phages have encouraged many companies to invest in the production of phage products (Table 2). In the context of food safety, ListShieldTM (formely known as LMP-102TM) was the first phage-based preparation approved by the FDA and the Environmental Protection Agency, and has also been approved in Canada and Israel. The manufacturer of ListShield is Intralytix, Inc. (Baltimore, MD, USA). They are a company specializing in the development and production of phage-based products for food safety. This product is composed of a mixture of six bacteriophages and is intended to be used in food processing plants as a decontaminant of surfaces and equipment as well as for treating foods with a high risk of *L. monocytogenes* contamination. Listex^TM^ P100 is based on bacteriophage P100 and has been approved as a clean label processing aid in several countries, including USA, Canada, Australia, New Zealand, Switzerland, Israel, Norway and the Netherlands. The manufacturer of Listex P100 is Micreos Food Safety, a Dutch company, recently rebranded as Phageguard (Wageningen, The Netherlands). They specialize in phage-based products, and Listex P100 is one of their key offerings. ListexTM P100 has been shown to reduce *L. monocytogenes* in fish [130], meat [131,132], vegetables, fresh fruits and fruit juices [133]. The bacteriophage product AgriphageTM, produced by OmniLytics Inc. (Sandy, UT, USA) to treat bacterial point diseases on crops, was the first phage product officially approved for use in agriculture by US regulators [134]. In 2007, the FDA approved the use of anti-*E. coli* and anti-*Salmonella* phage preparations, produced by OmniLytics Inc., to decontaminate live animals prior to slaughter [135]. The use of phages anti-foodborne bacterial pathogens was reviewed by [136], but showed variable success. Treatment of systemic salmonellosis in poultry has failed unless bacteriophages were given immediately after the bacteria. Reduction in intestinal colonization, aimed at reducing the entry of this organism into the human food chain, has also not been successful [137].

However, in 2010, the Korean company CheilJedang Corporation (Seoul, Republic of Korea) introduced BioTector, a bacteriophage product intended to reduce Salmonella levels in poultry (Figure 1; Table 2). EcoShield^TM^ (Intralytix Inc.) received regulatory approval in 2011 for use against *E. coli* O157:H7 on red meat to be ground in burgers, removing 95–100% of contaminants [138]. CUSTUS^®^YRS is a commercialized product by a Norwegian company (ACD Pharma; Leknes, Norway), recently approved, also as a processing aid, to control *Yersinia ruckeri* (*Y. ruckeri*), a very problematic salmon pathogen in aquaculture. CUSTUS^®^YRS is a bacteriophage product used in aquaculture to control the bacteria *Y. ruckeri*, which causes yersiniosis in fish. ACD Pharma is a Norwegian company specializing in developing bacteriophage-based solutions for aquaculture. They were the first to introduce a bacteriophage product for aquaculture use. There is also very marked interest in the poultry industry, which relies on cocktails against *Salmonella* or *Clostridium perfringens* (*C. perfringens*) [139]. SalmoFresh™ was an effective biocontrol intervention to reduce *Salmonella* on romaine lettuce, mung bean sprouts and seeds [140]. The manufacturer of SalmoFresh™ is Intralytix, Inc. SalmoFresh™ is an antimicrobial preparation used to control *Salmonella* in food.

The high specificity of certain phages for their host bacteria, along with their rapid replication compared to host cell division, makes them well suited for fast detection applications. In fact, several commercial assays have been developed that utilize native phage propagation, and the range and formats of these phage-based detection systems continue to expand. The first commercially test was the FASTPlaqueTB^TM^ assay for the detection of *M. tuberculosis* in human sputum samples by a UK-based company, Biotec Laboratories (Ipswich, UK) [141]. A rapid assay has also been developed to identify methicillin-resistant *S. aureus* (MRSA). The MicroPhage MRSA screening test detects growth of a specific phage following infection of the target cell using antibody capture, and results are available within 5 h.

PHaGElab/Chile is a global biotech company with over 14 years of experience in partnering with poultry and livestock producers to develop tailor-made antibacterial solutions. The company targets and eliminates specific bacterial pathogens through an artificial intelligence-powered streamlined sample protocol to ensure long-term farm health by providing fast, precise diagnosis and solution development. INSPEKTOR^®^ is developed by PHaGElab. This product based on bacteriophages reduced *Salmonella* load in some Brazilian commercial chicken farms, being significant on control of Minnesota and Heidelberg serovars, the most prevalent detected in poultry in that country. Moreover, this study highlights that the INSPEKTOR’s application could be the largest application of bacteriophages known to date in real poultry production systems, which also demonstrated the high effectiveness of bacteriophages therapy as an alternative or a complement of routine antibiotics use [142].

SciPhage is a phage technology company offering an alternative to antibiotics in human and animal health for the control of bacterial infections and plant health in environments contaminated by bacteria. SalmoFREE^®^ includes a service that diagnoses the level of *Salmonella* contamination in chicken farms and laying hens and is integrated into the application of the SalmoFree product. Currently, the product is designed to be supplied in drinking water for production birds. SalmoFREE^®^ is a phage solution that may constitute an efficient prevention measure to avoid food poisoning outbreaks associated with *Salmonella*. Overall, [143] demonstrated that SalmoFREE^®^ does not affect the animals nor the production parameters, demonstrating its safety for broilers. This product also contributes to the reduction in the presence of *Salmonella*, when it is used in multiple doses.

#### 3.4.2. Water Treatment

Waterborne bacterial agents (coliforms and other *Enterobacteriaceae*) in wastewater, surface water, water effluent, in rivers and in treated wastewater intended for irrigation remain a public health problem. This issue is important not only because of the environmental damage, morbidity, and mortality these pathogens cause, but also due to the substantial costs associated with wastewater disinfection using physical and chemical methods in treatment plants. A multitude of studies have shown that conventional wastewater treatment methods are insufficient for the definitive elimination of pathogenic bacteria which are vectors for the spread of diseases [2,144,145]. Bacteriophages are proposed as indicators of bacterial pathogens and as an alternative biological method for wastewater treatment [145,146].

Numerous studies report applications of phages as indicators or tracers of the presence of bacteria in wastewater treatment systems [147,148,149]. These approaches could help predict bacterial contamination in wastewater, allowing the use of other disinfection methods to treat pathogenic bacteria. Others have suggested using phages directly in wastewater treatment [2,147,150]. In this regard, many studies have proposed the use of specific phages as an ecological approach for the biological control of sludge and the treatment of filamentous bacteria in activated sludge process (ASP) systems [151,152,153]. Several lytic phages could also be exploited as a biocontrol of filamentous bacteria, which could lead to reduced foaming in the ASP processing plant [147,154,155]. These studies concluded that the development of sludge treatment using phages can provide long-term, cost-effective control of potentially pathogenic bacteria (e.g., *E. coli* and *Salmonella*) [147,156].

## 4. Conclusions and Future Outlook

Bacteriophages have exhibited significant utility across diverse scientific domains since their discovery. Notably, they have emerged as crucial agents in combating bacterial infections, ensuring food safety, and mitigating the challenges posed by antimicrobial-resistant strains of bacterial pathogens, particularly in the context of increasing resistance rates. Bacteriophages offer a highly promising and alternative therapeutic approach to tackle bacterial diseases, while also showcasing their efficacy as tools in vaccine delivery systems, modulation of bacterial populations, and diagnostic strategies. The multifaceted applications of bacteriophages underscore their potential as invaluable tools in addressing bacterial infections and driving scientific advancements in various fields.

The utilization of bacteriophages in the control of foodborne pathogens is a subject of active exploration. These entities are being investigated as biocontrol agents within food processing environments, serving to combat the presence of pathogens. Additionally, bacteriophages show promise as antimicrobial agents for food preservation purposes, effectively inhibiting the growth and proliferation of harmful bacteria. Furthermore, their application as bioactive coatings on food packaging materials presents a novel avenue to enhance food safety and extend shelf life. One of the advantages of using bacteriophages in food control is their ability to specifically target and kill foodborne pathogens without affecting non-pathogenic bacteria or other components of food products. This targeted approach may help reduce the use of chemical antimicrobials in food production and processing, which can have potential implications for food safety, quality, and consumer preferences. Nevertheless, for example, in food packaging, the use of phages is still in the early stages of research and development, and there is still much to be learned about their efficacy and safety. It will be important for further research to demonstrate the long-term stability and efficacy of phages in food packaging, as well as their safety for consumers and the environment.

With regard to the application of phages as therapy, it is still considered an experimental treatment, and regulatory approval for its widespread clinical use varies by country. There are challenges and limitations to bacteriophage therapy, including the need for careful selection and characterization of bacteriophages, potential issues with bacterial resistance to bacteriophages, and the lack of standardized protocols and regulatory frameworks. Nonetheless, bacteriophage therapy holds promise as a potential treatment option for bacterial infections, and ongoing research continues to explore its safety, efficacy, and potential applications in clinical settings. Further studies and clinical trials are needed to fully understand the safety and effectiveness of bacteriophage therapy and to establish standardized protocols for its clinical use. In fact, the use of phages has many limits due to fears related to their use (genetic recombination for example) and legislation, but on the other hand, the application of enzymes derived from phages may become more attractive than the use of phages themselves. Therefore, further studies should also focus on the exploitation of phage enzymes for use as antibacterial agents.

In relation to wastewater treatment, the use of phages as a targeted approach may help reduce the use of chemicals and antibiotics, which can have environmental and public health implications. Additionally, bacteriophages can replicate in the presence of their target bacteria, potentially allowing for their self-replication and sustained efficacy in treating bacterial pathogens in wastewater. The principal challenges include the need for careful selection and characterization of bacteriophages, potential issues with bacterial resistance to bacteriophages, and the need for proper monitoring and regulation of bacteriophage use in wastewater treatment to ensure safety and effectiveness.

## Figures and Tables

**Table 2:** Commercial phage-based products. The information presented in this table reflects data verified from publicly available and valid sources at the time of revision. Product availability, company ownership, and web addresses are subject to change over time. As product names may also vary due to rebranding or updates, and some scientific studies refer to former or alternative names, the most commonly used or currently recognized names have been adopted here for consistency.

Product	Use	Company	Website
AgriPhage^TM^	Used on foods and crops targeting *Xanthomonas* pv. vesicatoria, *Pseudomonas* pv. tomato	OmniLytics (Sandy, UT, USA)	https://omnilytics.com/
BioTector	Feed additive for controlling *Salmonella* in poultry	CheilJedang Corporation (Seoul, Republic of Korea)	https://cjbio.net/en/
CUSTUS^®^_YRS_	Biocontrol against *Yersinia ruckeri* (aquaculture)	ACD Pharma (Leknes, Norway)	https://acdpharma.com/
EcoShield^TM^	Control of *E. coli* O157:H7 in foods and processing environments	Intralytix (Columbia, MD, USA)	https://www.intralytix.com/
FASTPlaque-Response^TM^	Rapid detection of rifampicin resistance in *Mycobacterium tuberculosis* from sputum	Biotech Laboratories Ltd. (Ipswich, UK)	No longer available; acquired by Lab21 Ltd. (Cambridge, UK), then by Novacyt Group (Chandlers Ford, UK)
FASTPlaqueTB^TM^	Rapid detection of *Mycobacterium tuberculosis* in sputum	Biotech Laboratories Ltd. (Ipswich, UK)	No longer available; acquired by Lab21 Ltd. (Cambridge, UK), then by Novacyt Group (Chandlers Ford, UK)
INSPEKTOR^®^	Reducing *Salmonella* load in poultry farms	PHaGElab (Santiago, Chile)	https://phage-lab.com/
LISTEX^TM^ P100	Food processing aid to eliminate *Listeria monocytogenes* strains on food products	Micreos Food Safety, rebranded PhageGuard (Wageningen, The Netherlands)	https://phageguard.com/
ListShield^TM^	Control of *Listeria monocytogenes* in foods and processing environments	Intralytix (Columbia, MD, USA)	https://www.intralytix.com/
SalmoFree^®^	Feed additive for controlling Salmonella in poultry	SciPhage (Bogota, Colombia)	https://sciphage.com/
SalmoFresh^TM^	Designed to control *Salmonella enterica* in ready-to-eat foods, surfaces and food contact environments	Intralytix (Columbia, MD, USA)	https://www.intralytix.com/

## Data Availability

No new data were created or analyzed in this study. Data sharing is not applicable to this article.

## Abbreviations

The following abbreviations are used in this manuscript:

AMS	Antimicrobial Stewardship
AMR	Antimicrobial resistance
ICTV	International Committee on Taxonomy of Viruses
dsDNA	Double-Stranded Deoxyribonucleic Acid
ssRNA	Single-Stranded Ribonucleic Acid
ssDNA	Single-Stranded DNA
dsRNA	Double-Stranded RNA
R-M	Restriction–Modification
R&D	European Research & Development
*E. coli*	*Escherichia coli*
*P. aeruginosa*	*Pseudomonas aeruginosa*
US	United States
FDA	Food and Drug Administration
EFSA	European Food Safety Authority
CRISPR-Cas	Clustered Regularly Interspaced Short Palindromic Repeats Associated Protein Cas system
ESBL	Extended-Spectrum Beta-Lactamase
*K. pneumoniae*	*Klebsiella pneumoniae*
*L. monocytogenes*	*Listeria monocytogenes*
VLPs	Virus-Like Particles
COVID-19	Coronavirus Disease of 2019
HPV	Human Papillomavirus
*S. aureus*	*Staphylococcus aureus*
*M. abscessus*	*Mycobacterium abscessus*
PFGE	Pulsed Field Gel Electrophoresis
*S. typhimurium*	*Salmonella typhimurium*
*S. Enteritidis*	*Salmonella enteritidis*
*Y. ruckeri*	*Yersinia ruckeri*
*C. perfringens*	*Clostridium perfringens*
